# Effects of oxaliplatin on mouse myenteric neurons and colonic motility

**DOI:** 10.3389/fnins.2013.00030

**Published:** 2013-03-12

**Authors:** Linah Wafai, Mohammadali Taher, Valentina Jovanovska, Joel C. Bornstein, Crispin R. Dass, Kulmira Nurgali

**Affiliations:** ^1^College of Health and Biomedicine, Victoria UniversityMelbourne, VIC, Australia; ^2^Department of Physiology, University of MelbourneParkville, VIC, Australia

**Keywords:** chemotherapy, enteric neuropathy, oxaliplatin, neurotoxicity, nitric oxide synthase (NOS), colon motility

## Abstract

Oxaliplatin, an anti-cancer chemotherapeutic agent used for the treatment of colorectal cancer, commonly causes gastrointestinal side-effects such as constipation, diarrhoea, nausea, and vomiting. Damage to enteric neurons may underlie some of these gastrointestinal side-effects, as the enteric nervous system (ENS) controls functions of the bowel. In this study, neuronal loss and changes to the structure and immunoreactivity of myenteric neuronal nitric oxide synthase (nNOS) neurons were examined in colonic segments from mice following exposure to oxaliplatin *ex vivo* and following repeated intraperitoneal injections of oxaliplatin over 3 weeks *in vivo*, using immunohistochemistry and confocal microscopy. Significant morphological alterations and increases in the proportion of NOS-immunoreactive (IR) neurons were associated with both short-term oxaliplatin exposure and long-term oxaliplatin administration, confirming that oxaliplatin causes changes to the myenteric neurons. Long-term oxaliplatin administration induced substantial neuronal loss that was correlated with a reduction in both the frequency and propagation speed of colonic migrating motor complexes (CMMCs) *in vitro*. Similar changes probably produce some symptoms experienced by patients undergoing oxaliplatin treatment.

## Introduction

Oxaliplatin, a third-generation platinum drug, has become one of the first-line therapies used in the treatment of metastatic colorectal cancer (de Gramont et al., 2000; Petrioli et al., 2008). Commonly administered in combination with 5-fluorouracil and leucovorin, oxaliplatin has been shown to improve response rates and survival of patients (André et al., 2004), and has anti-tumor activity against some cancer cell lines resistant to first-generation (cisplatin) and second-generation (carboplatin) platinum drugs (Machover et al., 1996; Louvet et al., 2002; Raymond et al., 2002). Although oxaliplatin is widely used clinically, there remains a poor understanding of its mechanisms of action. Oxaliplatin is known to be activated by non-enzymatic hydrolysis and displacement of the oxalate ligand, which forms highly reactive compounds that react with proteins, RNA, and DNA, specifically nuclear DNA, whereas mitochondrial DNA is less affected (Woynarowski et al., 1998; Kweekel et al., 2005). Like cisplatin, oxaliplatin preferentially binds to guanine clusters to mainly form intrastrand crosslinks (Woynarowski et al., 1998; Hector et al., 2001; Fuertes et al., 2003; Kweekel et al., 2005; Wang and Lippard, 2005). The platinum-DNA adducts formed are believed to inhibit DNA replication and transcription and to induce apoptosis or necrosis in cancer cells and rapidly dividing cell lines (Fuertes et al., 2003; Kweekel et al., 2005).

A frequent complication associated with chemotherapeutic agents is toxicity, which poses an imperative obstacle in the treatment of cancer patients, as doses of chemotherapy are consequently limited. Oxaliplatin toxicity is distinct from that produced by other platinum drugs, with very little hematotoxicity and no renal toxicity (de Gramont et al., 2000). The most common adverse effects due to oxaliplatin-induced toxicity include peripheral neurotoxicity (acute and persistent), ototoxicity, weight loss, fatigue, and gastrointestinal symptoms such as nausea, vomiting, constipation, and diarrhoea (Visovsky, 2003; Leonard et al., 2005). The gastrointestinal symptoms often appear shortly after treatment (acute toxicities) or long after treatment (late toxicities), with a subsequent period of toxicity ranging from months to years (Di Fiore and Van Cutsem, 2009). Although the gastrointestinal symptoms are widely acknowledged as adverse effects of platinum-based chemotherapeutic drugs, research into their underlying mechanism is scarce. It is known that platinum compounds have a high affinity for the peripheral nervous system (PNS) (Cavaletti et al., 2001; McDonald et al., 2005), and there is a possibility that these compounds may also react within the enteric nervous system (ENS), which is the largest and most complex subdivision of the PNS.

The ENS is located within the wall of the gastrointestinal tract and responsible for its physiological functions such as secretion, blood flow, and motility (Furness et al., 2008; Burzynski et al., 2009). Damage to enteric neurons, or changes in their electrophysiological properties, results in altered and deficient physiological functions of the gut which has been demonstrated in studies of intestinal inflammation (Linden et al., 2003; Lomax et al., 2007; Nurgali et al., 2007, 2011). Deficient physiological functions have also been demonstrated as a result of damage to neurons in the ENS of rats following long-term cisplatin administration. Marked reductions in both gastrointestinal transit rate and colonic motor activity were observed, alongside a significant decrease in the number of neurons per ganglion, and an increase in the proportion of nitric oxide synthase-immunoreactive (NOS-IR) neurons in the myenteric plexus of the rat colon (Vera et al., 2011). Intriguingly, an upregulation of neuronal NOS (nNOS) has also been reported in the spinal cord of rats following repeated oxaliplatin administration (Mihara et al., 2011).

The nNOS enzyme is responsible for the synthesis of the free-radical gas nitric oxide (NO), which mediates inhibitory neuromuscular transmission within the gastrointestinal tract and may also have roles in the enteric neural circuits themselves (Yuan et al., 1995; Dickson, 2007; Bornstein et al., 2010). Changes to neurons expressing nNOS have been linked to a number of enteric neuropathies, some of which include colonic dysfunction associated with diabetes and ischemia of the intestine (Furness, 2006; Rivera et al., 2011).

In this study, we investigated the effects of both *ex vivo* tissue culture with oxaliplatin, and the effects of short- and long-term repeated *in vivo* oxaliplatin administration on the myenteric neurons of the mouse colon. In particular, we aimed to determine the nature of oxaliplatin-induced changes to the myenteric neurons by examining neuronal loss and nNOS expression. Moreover, we studied the effects of *in vivo* oxaliplation administration on spontaneous motor activity of the mouse colon.

## Materials and methods

### Animals

Male Balb/c mice aged 5–8 weeks (18–25 g) were used in this study. Mice were supplied from the Animal Resources Centre (WA, Australia). Animals were kept on a 12 h light and dark cycle at ~22°C with free access to food and water. The mice were allowed to acclimatize for at least 1 week following arrival.

All procedures in this study were approved by the Victoria University Animal Experimentation Ethics Committee and performed in accordance with the guidelines of the National Health and Medical Research Council.

### *Ex vivo* tissue culture

Control Balb/c mice were euthanized by cervical dislocation on the day of the experiment and the distal colon segments were removed for *ex vivo* tissue culture with oxaliplatin (Sigma-Aldrich, Australia). Tissues were grouped into (1) control, (2) low concentration oxaliplatin (10 nM), and (3) high concentration oxaliplatin (10 μM). All colon segments were pinned onto a silicon base dish and incubated in oxygenated (95% oxygen, 5% carbon dioxide) Dulbecco's Modified Eagle's Medium (DMEM) (Sigma-Aldrich, Australia) containing 10% foetal bovine serum (Invitrogen, Australia), 1% antibiotic/antimycotic solution (comprising penicillin, streptomycin, and amphotericin) (complete DMEM) (Sigma-Aldrich, Australia), along with the respective dose of oxaliplatin for a period of 3 and 24 h. During incubation, the dishes containing the distal colon segments were kept constantly shaking on a Unimax-1010 platform shaker (Heidolph, Germany) under sterile conditions at 37°C using an Incubator-1000 (Heidolph, Germany). Following incubation, tissues were then cut open along the mesenteric border, maximally stretched and pinned mucosa up into the same dish, before being fixed overnight at 4°C with Zamboni's fixative containing 2% formaldehyde and 0.2% picric acid.

### *In vivo* oxaliplatin injections

Mice were grouped into (1) oxaliplatin-treated and (2) sham-treated cohorts. The mice from group 1 received intraperitoneal injections of oxaliplatin (3 mg/kg) three times a week with 26 gauge needles, and mice from group 2 were injected with sterile water. Mice were euthanized by cervical dislocation at the end of the experimental period (3, 7, 14, and 21 days). Segments of the distal colon were removed and placed in oxygenated phosphate-buffered saline (PBS, pH 7.2) containing the L-type Ca^2+^ channel blocker nicardipine (3 μM) for 20 min before being cut open along the mesenteric border, maximally stretched and pinned into a silicon base dish. Tissues were then fixed with Zamboni's fixative overnight at 4°C.

### Immunohistochemistry

Fixed tissues were washed three times for 10 min each with dimethyl sulfoxide (DMSO) (Sigma-Aldrich, Australia) followed by three 10 min washes with PBS. Tissues were then dissected to expose the myenteric plexus by removing the mucosa, submucosa, and circular muscle layers. Tissues were incubated for 1 h at room temperature with 10% normal donkey serum (Millipore, MA, USA). Myenteric neurons were double labeled in these wholemount preparations with primary antibodies raised in different species: anti-β-Tubulin class III, TuJ1 (Rabbit, 1:2000, Abcam, MA, USA), anti-nNOS (Goat, 1:500, Novus Biologicals, CO, USA) overnight at 4°C. Tissues were washed three times for 10 min with PBS before incubation with species-specific secondary antibodies labeled with different flurophores: Donkey anti-rabbit Alexa 488 and 594 (1:200, Invitrogen, VIC, Australia), Donkey anti-goat FITC (1:200, Jackson Immunoresearch Laboratories, PA, USA) for 2 h at room temperature. Tissues were given a further three 10 min washes with PBS, followed by a 2 min incubation with the fluorescent nucleic acid stain 4′-6-diamidino-2-phenylindole (DAPI) (14 nM) (Invitrogen, Australia). Tissues received one final 10 min PBS wash before being mounted onto glass slides with fluorescent mounting medium (DAKO, Australia).

### Imaging

Three-dimensional (z-series) images of the wholemount preparations were taken using an Olympus FluoView FV1000 confocal laser scanning microscope (Olympus, Australia). Fluorophores were visualized using excitation filters for Alexa 594 and Rhodamine Red (excitation wavelength 559 nm), Alexa 488 and FITC (excitation wavelength 473 nm), and DAPI (excitation wavelength 405 nm). Z-series images were taken at a stepsize of 1.75 μm (1600 × 1200 pixels).

### Neuronal cell counting

The wholemount preparations were observed under a fluorescent microscope Olympus BX53 (Olympus, Australia). Each preparation was analysed by randomly capturing four images at 20 × magnification using a DP72 camera and processed by cellSens Standard 1.4.1 software (Olympus, Australia). The total number of neurons per 2 mm^2^ area was quantified by counting TuJ1-positive cells with round or oval shape nuclei counterstained with DAPI within the ganglia. NOS immunoreactivity was quantified by counting the number of nNOS positive neurons within the same area. Data were obtained from three animals per time point (*ex vivo*: 3 and 24 h; *in vivo*: 3, 7, 14, and 21 days).

### Neuronal measurement

The total area of individual NOS-IR neurons (μm^2^), including dendrites, was measured by tracing neuronal profiles using Image J software (NIH, MD, USA). The cohort size was 30 neurons per animal. Data were obtained from three animals per time point (3, 7, 14, and 21 days) for both oxaliplatin-treated and sham-treated groups.

### Colonic motility experiments

The entire colon (5–6 cm) was removed from control, Day 14 sham, and Day 14 oxaliplatin-treated animals (*n* = 5 per each group) and set up to allow spatiotemporal maps to be made of spontaneous motor patterns *in vitro* (Roberts et al., 2007, 2008). Briefly, the colon was left in physiological saline (composition in mM: NaCl 118, KCl 4.6, CaCl_2_ 3.5, MgSO_4_ 1.2, NaH_2_PO_4_ 1, NaHCO_3_ 25, d-Glucose 11, oxygenated with 95% O_2_ and 5% CO_2_), until the faecal pallets were expelled. The empty colon was cannulated at both ends and set up horizontally in organ bath chambers superfused with oxygenated physiological saline and kept at a constant temperature of 35°C. The oral cannula was connected to a reservoir-containing oxygenated physiological saline. The intraluminal pressure of the segment was maintained at +2 cm H_2_O, by adjusting the height of a reservoir connected to the oral cannula. The anal end was connected to an outflow tube that provided a maximum of 2 cm H_2_O back-pressure. Contractile activity of each segment was recorded with a Logitech Quickcam pro camera positioned 7–8 cm above the preparation. Following a 30 min equilibration period, four 15 min videos were captured. Videos were then used to construct spatiotemporal maps of the diameter at each point along the segment using in house software (Gwynne et al., 2004). Spatiotemporal maps were used to quantify the frequency and propagation speed of colonic migrating motor complexes (CMMCs), defined as constrictions that propagate for at least 50% of the length of the colon (Roberts et al., 2007, 2008).

### Statistical analysis

Data were assessed using a Two-Way ANOVA followed by a Bonferroni's *post-hoc* test. Analyses were performed using Graph Pad Prism (Graph Pad Software Inc., CA, USA). All data are presented as mean ± standard error of the mean (SEM). Value differences were considered statistically significant at *P* < 0.05.

## Results

### Effects of low and high oxaliplatin concentrations on myenteric neurons in tissue culture

To determine the effects of oxaliplatin on myenteric neurons at various concentrations, *ex vivo* distal colon segments were cultured with either a low (10 nM) or a high concentration (10 μM) of oxaliplatin for 3 and 24 h.

**Figure 1:**
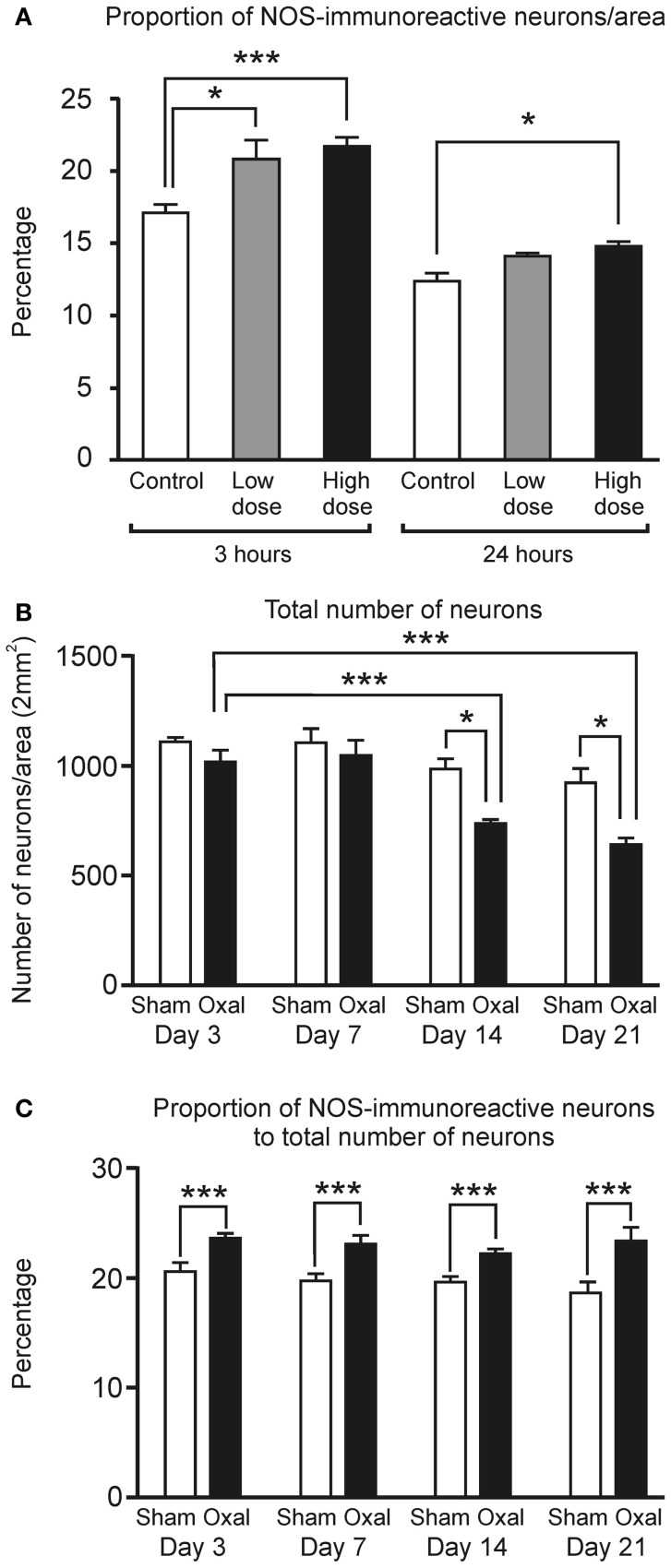
***In vitro* and *in vivo* effects of oxaliplatin on the myenteric neurons in the mouse distal colon. (A)** A significant increase in the proportion of NOS-IR neurons per area (2 mm^2^) within the myenteric plexus of the distal colon cultured with a high concentration of oxaliplatin compared to the control at both 3 (^***^P < 0.001) and 24 h (^*^P < 0.05) and a low concentration of oxaliplatin at 3 h of tissue culture (^*^P < 0.05). **(B)** A significant decrease in the total number of myenteric neurons at both Day 14 and Day 21 following *in vivo* oxaliplatin administration compared to the sham-treated at the same time points (^*^P < 0.05) and Day 3 oxaliplatin-treated mice (^***^P < 0.001). **(C)** Proportion of NOS-immunoreactive neurons per area (2 mm^2^) within the myenteric plexus of the distal colon was significantly increased at all four time-points (Days 3, 7, 14, and 21) following *in vivo* oxaliplatin administration compared to the sham-treated mice (^***^P < 0.001). All data represent the mean ± SEM.

After 3 h in culture, a notable increase in the proportion of NOS-IR myenteric neurons per area (2 mm^2^) was detected in the tissues incubated with both the low dose of oxaliplatin (20.7 ± 1.4%, *P* < 0.05) and the high dose of oxaliplatin (21.7 ± 0.7%, *P* < 0.001) in comparison to the control group (17.1 ± 0.5%) (Figure 1A). A significantly increased proportion of NOS-IR myenteric neurons was also seen following 3 h in culture with a high concentration of oxaliplatin per 2 mm^2^ (control: 127.3 ± 13.9; high dose: 170.7 ± 16.2, *P* < 0.05). After 24 h in culture, the high dose oxaliplatin group also displayed a higher proportion of NOS-IR myenteric neurons than controls (control: 12.4 ± 0.5%; high dose: 14.8 ± 0.3%, *P* < 0.05) (Figure 1A).

### Effects of repeated *in vivo* oxaliplatin administration on myenteric neurons

**Figure 2:**
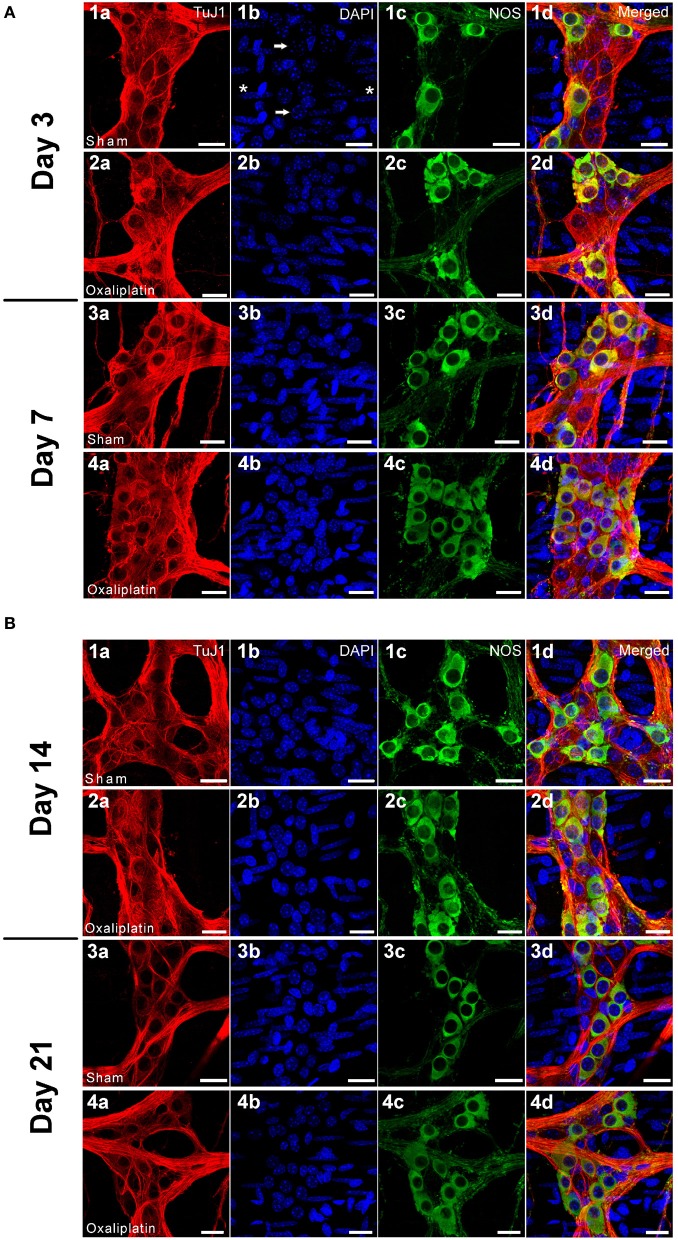
**(A)** Wholemount preparations of the myenteric neurons in the distal colon segments following 3 (2a–2d) and 7 (4a–4d) days of repeated *in vivo* oxaliplatin administration compared to sham-treated animals at 3 (1a–1d) and 7 (3a–3d) days. Myenteric ganglia and neurons labeled with β-Tubulin Tuj1 (red), nuclei labeled with DAPI (blue) (arrows) can be seen within the ganglion (1b). Smooth muscle cells (asterisks) are also labeled with DAPI (1b). Significant increase in the proportion of NOS neurons (green) can be seen at both Days 3 and 7 after oxaliplatin treatment. Scale bar = 20 μm. **(B)** Wholemount preparations of the myenteric neurons in the distal colon segments following 14 (2a–2d) and 21 (4a–4d) days of repeated *in vivo* oxaliplatin administration compared to sham-treated animals at 14 (1a–1d) and 21 (3a–3d) days. The reduction in total number of neurons and increase in the proportion of NOS neurons was found at both Days 14 and 21 after oxaliplatin treatment. Scale bar = 20 μm.

To determine whether the changes induced by oxaliplatin in the *ex vivo* tissue culture also occur in *in vivo*, the effects of repeated short- and long-term oxaliplatin administration were examined (Figures 2A,B).

The total number of myenteric neurons was significantly reduced in the oxaliplatin-treated group when compared to the sham-treated group at Day 14 (sham-treated: 970 ± 46; oxaliplatin-treated: 726 ± 15, *P* < 0.05) and at Day 21 (sham-treated: 907 ± 67; oxaliplatin-treated: 629 ± 30, *P* < 0.01). A comparison within the oxaliplatin-treated group alone also shows a substantial reduction in the number of neurons from Day 3 (1007 ± 50) to Days 14 (726 ± 15, *P* < 0.001) and 21 (629 ± 30, *P* < 0.001) (Figure 1B).

Consistent with the *ex vivo* tissue culture, wholemount preparations of the distal colon from *in vivo* oxaliplatin-treated mice displayed a substantial increase in the proportion of NOS-IR myenteric neurons at all four time points following treatment (Day 3: 23.7 ± 0.4%; Day 7: 23.2 ± 0.7%; Day 14: 22.3 ± 0.4%; and Day 21: 23.6 ± 1.2%) in comparison to the sham-treated group (Day 3: 20.6 ± 0.8%; Day 7: 17.8 ± 0.7%; Day 14: 19.7 ± 0.5%; and Day 21: 18.7 ± 1.1%, *P* < 0.05) (Figure 1C).

### Morphological changes in NOS-immunoreactive neurons

The morphology of the NOS-IR myenteric neurons was examined in the *in vivo* sham-treated and oxaliplatin-treated groups to determine if oxaliplatin administration produced changes to the neuronal shape and size.

**Figure 3:**
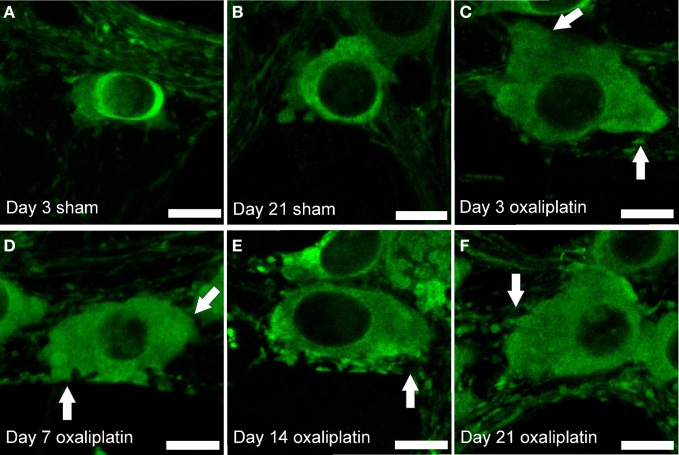
**Morphological changes in myenteric NOS-IR neurons following repeated intraperitoneal administration of oxaliplatin.** Neurons from mice receiving oxaliplatin from Days 3 to 21 **(C–F)** display an increase in the soma size and prominent, slightly distorted dendrites (arrows) when compared to the neurons from the sham-treated mice **(A,B)**. Scale bar = 10 μm.

NOS-IR myenteric neurons in wholemount preparations of the distal colon from the oxaliplatin-treated mice appear to be larger, with more prominent and distorted dendrites at all four time-points (Day 3, 7, 14, and 21) in comparison to the sham-treated group (Figure 3).

**Figure 4:**
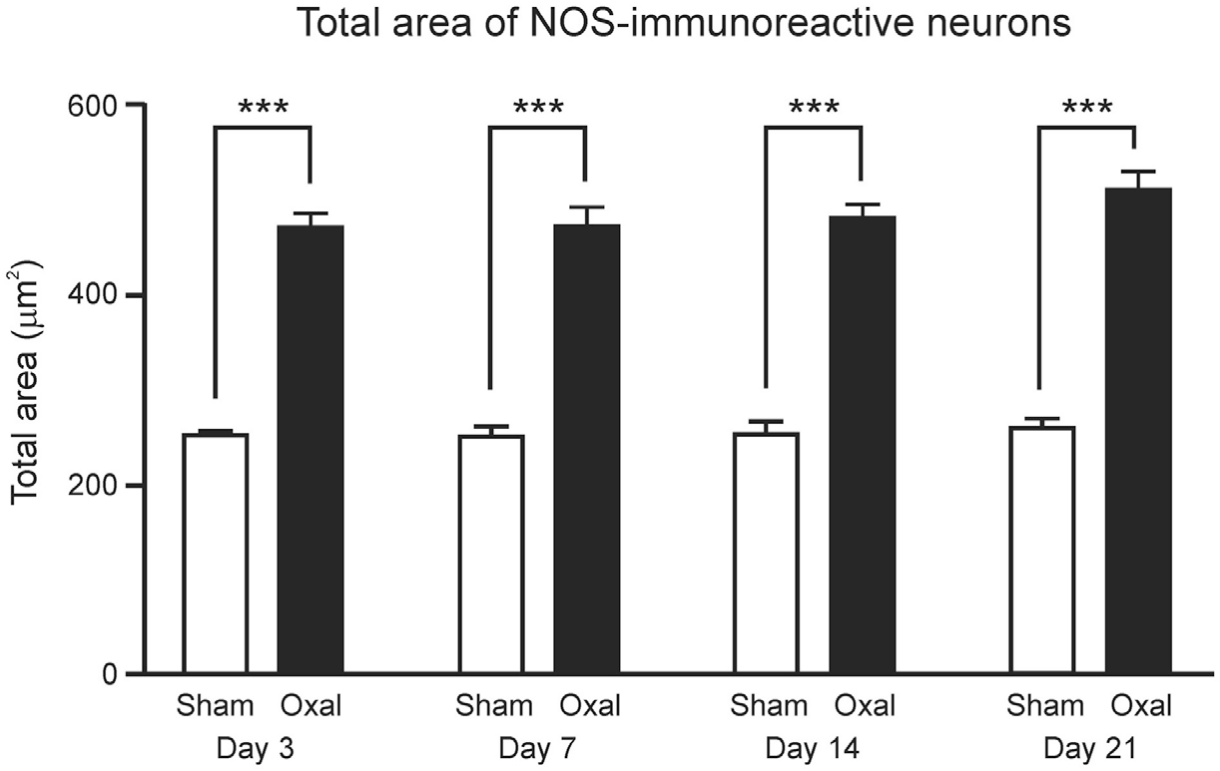
**Effects of repeated *in vivo* oxaliplatin administration on the total area of NOS-IR neurons (μm^2^) within the myenteric plexus of the distal colon segments.** Data represent the mean ± SEM. A significant increase in the total area of neurons immunoreactive to NOS was observed at all four time points (Day 3, 7, 14, and 21), following oxaliplatin administration in comparison to the sham-treated mice (^***^P < 0.001).

Measurements of the profile of NOS-IR neurons verified that total neuronal area, including dendrites, was significantly increased at all four time-points in the oxaliplatin-treated group (Day 3: 466 ± 17 μm^2^; Day 7: 470 ± 20 μm^2^; Day 14: 479 ± 14 μm^2^; and Day 21: 509 ± 20 μm^2^) when compared to the sham-treated group (Day 3: 251 ± 4 μm^2^; Day 7: 250 ± 11 μm^2^; Day 14: 253 ± 14 μm^2^; and Day 21: 260 ± 10 μm^2^, *P* < 0.001) (Figure 4).

### Changes in colonic motility

**Figure 5:**
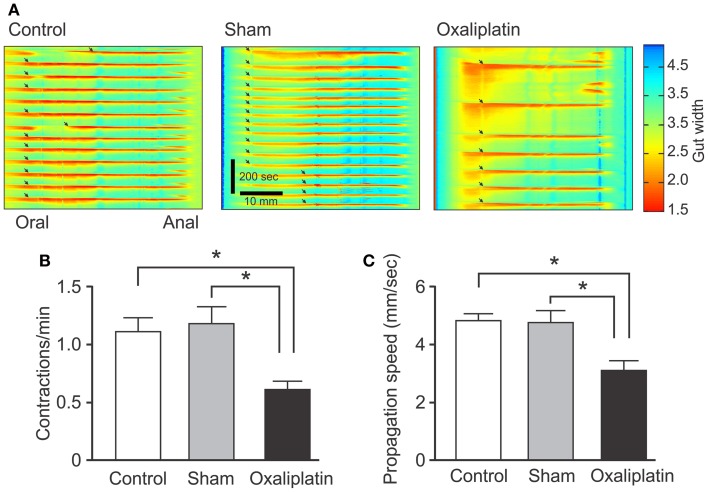
**Effects of *in vivo* oxaliplatin treatment on propulsive activity of the colon.** Spatiotemporal maps of the colon **(A)** from control, Day 14 sham-treated, and Day 14 oxaliplatin-treated mice. Anally propagating contractions that begin at the oral end of the preparation and continue to the anal end can be readily seen as red areas (arrows) separated by periods of quiescence. Frequency of contractions **(B)** and propagation speed **(C)** decreased significantly in the colon from Day 14 oxaliplatin-treated mice compared to both sham-treated and control groups (^*^P < 0.05 for all). Data represent the mean ± SEM.

To identify functional consequences of the anatomical changes produced by oxaliplatin, video-recordings of colonic motor activity were used to construct spatiotemporal maps of CMMCs, a repetitive stereotyped motor pattern characteristic of the isolated mouse colon (e.g., Roberts et al., 2007, 2008). Analysis of maps from Day 14 oxaliplatin-treated mice showed a significant decrease in both frequency (0.6 ± 0.07 per min) and propagation speed (3.1 ± 0.4 mm/s) of CMMCs compared to sham-treated (frequency of contractions: 1.2 ± 0.14 per min, *P* < 0.05; speed: 4.8 ± 4 mm/s, *P* < 0.05) and control groups (frequency of contractions: 1.1 ± 0.1 per min, *P* < 0.05; speed: 4.8 ± 0.3 mm/s, *P* < 0.05) (Figure 5).

## Discussion

This study is the first to examine the effects of oxaliplatin on the myenteric neurons in a mouse model of chemotherapy. Numbers and nNOS-IR of myenteric neurons were compared under two conditions: (1) *ex vivo* tissue culture with oxaliplatin for 3 and 24 h; and (2) repeated *in vivo* oxaliplatin administration into mice for up to 3 weeks. This allowed some neurotoxic effects of oxaliplatin on enteric neurons over short and extended periods of oxaliplatin administration to be evaluated and correlated with chronic oxaliplatin effects on colonic motility, *in vitro*.

Our major findings are consistent with an earlier study of the effects of cisplatin on rat enteric neurons (Vera et al., 2011). Like cisplatin in rats, oxaliplatin in mouse causes a proportional increase of NOS-IR neurons and a significant reduction in the total number of myenteric neurons. The decrease in numbers of neurons correlates with a reduction in CMMC frequency, something seen previously in mouse models of Hirschsprung disease (Roberts et al., 2008).

Dorsal root ganglion (DRG) neurons undergo apoptosis following exposure to oxaliplatin (Ta et al., 2006; Scuteri et al., 2009) and oxaliplatin accumulation in DRG neurons have been found (Holmes et al., 1998; Cavaletti et al., 2001; Ta et al., 2006). This has been proposed as the underlying cause of oxaliplatin-induced sensory neuropathy (Gill and Windebank, 1998). Platinum-based chemotherapeutic agents are designed to target and destroy proliferating cancer cells; but these agents also damage neurons, which are differentiated and non-proliferative. It has been suggested that platinum compounds may cause neurons to unsuccessfully re-enter the cell cycle (Gill and Windebank, 1998), as cell cycle protein expression is altered following exposure to cisplatin (Gill and Windebank, 1998; McDonald et al., 2005). Platinum-induced apoptosis has also been associated with the involvement of the mitogen-activated protein kinases (MAPK) family (Scuteri et al., 2009), which are responsible for regulating a number of cellular activities such as differentiation, proliferation, and cell death (Lewis et al., 1998). In particular, the extracellular signaling-regulated kinase (ERK 1/2) and p38, which have well-recognized roles in neuronal apoptosis, have displayed early activation in DRG neurons following exposure to both oxaliplatin and cisplatin (Takeda and Ichijo, 2002; Scuteri et al., 2009).

Although the mechanisms of platinum-induced neuronal death have yet to be studied in enteric neurons, it has been speculated that the death of sensory DRG neurons involves afferent neurons innervating the gastrointestinal tract (Vera et al., 2011). However, the extent of neuronal loss seen in our study suggests that oxaliplatin might also induce apoptosis of myenteric neurons which needs to be further studied. The death of enteric neurons is associated with impairment of colonic motility observed in our study and in a previous study with cisplatin (Vera et al., 2011), which may explain some gastrointestinal symptoms experienced by patients.

Further oxaliplatin-induced changes to myenteric neurons in this study included the significant increase in the proportion of NOS-IR neurons both *ex vivo* and *in vivo*. NOS, and in particular nNOS, seems to play a significant role in oxaliplatin-induced neurotoxicity. This is noteworthy, as nNOS is typically expressed by interneurons and inhibitory motor neurons that supply the circular and longitudinal muscles of the gastrointestinal tract (Bornstein et al., 2004). A recent study has shown that the repeated administration of oxaliplatin in the rat causes an upregulation of nNOS in the spinal cord (Mihara et al., 2011). This finding has been associated with the incidence of mechanical allodynia, the sensation of pain caused by a stimulus that would not normally produce pain, e.g., light touch or cold. The administration of both selective and non-selective NOS inhibitors prevented the pain behavior initially seen in rats following oxaliplatin administration.

The production of NO by nNOS depends on levels of cytoplasmic Ca^2+^. When cytoplasmic Ca^2+^ is elevated, nNOS is activated, possibly leading to excessive NO production, which causes cell damage (Yagihashi et al., 2000), and may account for the oxaliplatin-induced increase in NOS-IR neuron size seen in this study. This needs to be further investigated. Elevated cytoplasmic Ca^2+^ is also known to cause cytotoxic damage to neurons and can trigger apoptosis (Rivera et al., 2011).

Conversely, the increased proportion of NOS-IR neurons seen with oxaliplatin administration in our study could partly be due to the loss of other subpopulations of myenteric neurons. Therefore, these results could be due to a combination of both increases in cytoplasmic Ca^2+^ and free-radical NO, and the death of other neuronal subgroups within the ENS. It is also possible that NOS-IR neurons are capable of better survival following oxaliplatin administration than other subgroups of myenteric neurons, as nNOS neurons have been shown to remain quite resistant to toxicities in Alzheimer's disease, Huntington's disease, and vascular stroke (Gonzalez-Zulueta et al., 1998). Recent study in nNOS knockout mice has shown that NO produced by nNOS has protective effects on enteric neurons following intestinal ischemia/reperfusion injury (Rivera et al., 2012). Further studies need to be carried out investigating the mechanisms by which oxaliplatin-induced changes occur in the enteric neurons and determining which subpopulations are being affected. The effect of oxaliplatin on enteric neuron function also needs to be examined, including changes in the electrophysiological properties of neurons, neurotransmission, and ion channel functions.

### Conflict of interest statement

The authors declare that the research was conducted in the absence of any commercial or financial relationships that could be construed as a potential conflict of interest.
